# Seroprevalence of *Toxoplasma gondii* Infection in Sows in Hunan Province, China

**DOI:** 10.1155/2014/347908

**Published:** 2014-02-06

**Authors:** Ying Xu, Run-Cheng Li, Guo-Hua Liu, Wei Cong, Xiao-Xuan Zhang, Xing-Long Yu, Xing-Quan Zhu

**Affiliations:** ^1^State Key Laboratory of Veterinary Etiological Biology, Key Laboratory of Veterinary Parasitology of Gansu Province, Lanzhou Veterinary Research Institute, Chinese Academy of Agricultural Sciences, Lanzhou, Gansu 730046, China; ^2^College of Animal Science and Technology, Anhui Agricultural University, Hefei, Anhui 230036, China; ^3^College of Veterinary Medicine, Hunan Agricultural University, Changsha, Hunan 410128, China; ^4^College of Animal Science and Technology, Jilin Agriculture University, Changchun, Jilin 130118, China

## Abstract

*Toxoplasma gondii* infections are prevalent in animals and humans worldwide. Although the prevalence of *T. gondii* has been reported in many animals in China, little is known of *T. gondii* infection in sows. Antibodies to *T. gondii* in sows in Hunan province, subtropical China, were examined using indirect hemagglutination test (IHAT). Overall, 31.3% (373/1191) of the examined sows were seropositive for *T. gondii*. Among 11 representative regions of Hunan province, the seroprevalence ranged from 14.8% to 45.1%. In addition, the *T. gondii* seroprevalence was higher in summer (37.4%) and autumn (34.9%) than in spring (24.6%) and winter (23.9%). Regarding different antibody titers, the seroprevalence ranged from 1.8% (titer ≥ 1 : 1024) to 17.4% (titer = 1 : 64). The findings of the present investigation revealed the high seroprevalence of *T. gondii* in sows in Hunan province, China, which poses a potential risk for *T. gondii* infection in humans and animals in this province. Therefore, effective measures should be taken to prevent and control toxoplasmosis of pigs in this province. This is the first report of the comprehensive survey of *T. gondii* seroprevalence in sows in Hunan Province, subtropical China.

## 1. Introduction

*Toxoplasma gondii* is a protozoan parasite that infects a wide range of animals as well as humans and can cause toxoplasmosis [1]. It is prevalent in most areas of the world and up to one-third of the human population has been exposed to the parasite [1]. Humans get infected with *T. gondii* mainly by ingesting oocysts shed by cats or consuming undercooked meat containing parasite tissue cysts [2]. In addition, *T. gondii* in the pregnant women may be transmitted to foetus and cause severe consequences [3, 4]. Toxoplasmosis is usually subclinical or mild symptoms in health adults; however, it can be fatal in the very young and immunocompromised patients [1]. To date, toxoplasmosis continues to be a significant public health problem around the world, and no commercial vaccines are available, and treatment relies on chemical drugs [5, 6].

*T. gondii* infections are also very prevalent in all domestic animals and can cause major economic losses globally [7, 8]. Pigs are considered as an important intermediate host of *T. gondii*. Currently, China is the largest producer of pigs in the world, and Hunan province is the second largest pig-producing province. Since the 1980s, epidemiological studies of *T. gondii* infections in pigs have been conducted in many provinces [9–11]. But unfortunately, the majority of those reports were published in local Chinese journals, which are not readily accessible to international readers. In spite of the high prevalence of *T. gondii* reported in pigs in China, limited information is available for *T. gondii* infections in sows [12–14]. Moreover, a preliminary pilot survey showed that *T. gondii* may pose significant public health threats in this province [15].

The objective of the present investigation was to examine the *T. gondii* seroprevalence in sows in Hunan province, subtropical China. The results should provide a foundation for the execution of control strategies against *T. gondii* infection in pigs in this province and elsewhere.

## 2. Materials and Methods

### 2.1. Serum Samples

A total of 1191 blood samples were collected from sows in intensive farms in 11 representative administrative regions in Hunan province between January 2010 and August 2012 (Table 1). The sows were randomly selected, and one blood sample was collected from each animal. All the blood samples were labelled individually and cooled during transport to the laboratory at College of Veterinary Medicine, Hunan Agricultural University (Changsha, Hunan Province). Blood samples were then centrifuged at 1,000 g for 10 min, and serum was obtained, frozen, and stored at −20°C until use.

### 2.2. Serological Examination

*T. gondii* antibodies (IgG) in sows were tested by indirect hemagglutination test (IHAT) using a commercially marketed kit (NY/T 573-2002, Lanzhou Veterinary Research Institute, Chinese Academy of Agricultural Sciences) according to the manufacturer's recommendations and previous descriptions (e.g., [16, 17]). This kit has been extensively used for detecting specific antibodies to *T. gondii* in pigs, sheep, and other mammals in China for many years. The serum samples were identified as positive if agglutination reaction was seen in wells with dilutions of 1 : 64 or higher.

### 2.3. Statistical Analysis

The seroprevalence data were analyzed statistically using the PASW Statistics 18 (IBM Corporation, Somers, NY, USA), and 95% confidence intervals (CI) are also given. The value of *P* < 0.05 differences between levels within factors and interactions was considered to be statistically significant.

## 3. Results and Discussion

Antibodies against *T. gondii* were detected in 31.3% (373 of 1191) sows, with titers of 1 : 64 in 207, 1 : 128 in 74, 1 : 256 in 44, 1 : 512 in 27, and 1 : 1024 in 21, respectively. The *T. gondii* seroprevalence in sows from different regions ranged from 14.8% to 45.1% (Table 1), having statistically significant differences (*P* < 0.01). The seroprevalence of *T. gondii* in sows from 6 of the 11 representative administrative regions in Hunan province was more than 31.3% (average value), and the highest seroprevalence (45.1%) was in Zhuzhou (Table 1). The differences in seroprevalence may be related to stray cats because there is abundance of stray cats in Zhuzhou. *T. gondii* seroprevalence in sows was higher in summer (37.4%), followed by autumn (34.9%), but lower in spring (24.6%) and winter (23.9%) (Table 2), and these seroprevalences were statistically different (*P* < 0.01).

The present study revealed a high *T. gondii* seroprevalence (31.3%) in sows in Hunan province, China. These findings provide strong evidence that *T. gondii *infection is highly prevalent in sows in this region of China. This is likely due to the presence of large number of cats in public places and pig farms in Hunan province. *T. gondii* seroprevalence in sows in the present study was significantly higher than that in Yunnan (16.6%) [13] and Fujian provinces (14.4%) [14] but was lower than that in Guangdong province (36.9%) [12]. The differences in seroprevalence may be related to animal welfares, climates, and animal husbandry practices.

The present survey showed that seroprevalence of *T. gondii* was the highest in summer (37.4%), followed by autumn (34.9%), and was the lowest in winter (23.9%). This result suggested that *T. gondii* is prevalent all year round, with the peaks in summer and autumn. Hunan province has a subtropical moist monsoon climate with an average annual temperature of 16–18°C and a high humidity in summer and autumn, which is favorable for the survival of the oocysts [18, 19]. Other factors might have contributed to the seasonality in seroprevalence. The peaks of* T. gondii* seroprevalence in summer and autumn suggested that it is more important to control toxoplasmosis in both summer and autumn.

Toxoplasmosis can lead to abortion, stillborn, and mummification in pregnant sows [20]. However, the present dataset could not determine whether or not *T. gondii* infection can significantly increase the risk of abortion in the sow in Hunan province. Therefore, further studies are necessary to elucidate a potential effect of *T. gondii* on reproduction of sows. In addition, the ingestion of food or water that is contaminated with oocysts shed by cats is considered an important source of *T. gondii* infection in humans [21, 22]. The present and previous results [15] revealed the presence of *T. gondii* infection in pigs in this province, indicating contamination of the environment by *T. gondii* oocysts, posing a risk for human infection with *T. gondii*. So, further work is required to assess whether the soil and water in pig farms or other regions in Hunan province are also contaminated by *T. gondii* oocysts [23].

## 4. Conclusion

In summary, the present results indicated the high seroprevalence of *T. gondii* infection in sows in Hunan province, China; however, this severe situation received little attention in the past. Therefore, effective measures should be taken to prevent and control toxoplasmosis of pigs in this province.

## Figures and Tables

**Table 1:** Seroprevalence of *Toxoplasma gondii* infection in sows in Hunan Province, China.

Region	Number tested	Number positive	Prevalence (%)
Yongzhou	100	38	38
Changsha	196	29	14.8
Yueyang	106	43	40.6
Shaoyang	103	31	30
Loudi	81	14	17.3
Yiyang	114	36	31.6
Xiangxi	45	12	26.7
Hengyang	105	25	23.8
Chenzhou	100	44	44
Changde	108	41	38
Zhuzhou	133	60	45.1

Total	1191	373	31.3

**Table 2:** Seasonal seroprevalence of *Toxoplasma gondii* infection in sows in Hunan Province, China.

Seasons	Number tested	Number positive	Prevalence (%)
Spring	272	67	24.6
Summer	374	140	37.4
Autumn	327	114	34.9
Winter	218	52	23.9

Total	1191	373	31.3
